# Translating Attention-Deficit/Hyperactivity Disorder Rating Scale-5 and Weiss Functional Impairment Rating Scale-Parent Effectiveness Scores into Clinical Global Impressions Clinical Significance Levels in Four Randomized Clinical Trials of SPN-812 (Viloxazine Extended-Release) in Children and Adolescents with Attention-Deficit/Hyperactivity Disorder

**DOI:** 10.1089/cap.2020.0148

**Published:** 2021-04-16

**Authors:** Azmi Nasser, Alisa R. Kosheleff, Joseph T. Hull, Tesfaye Liranso, Peibing Qin, Gregory D. Busse, Welton O'Neal, Maurizio Fava, Stephen V. Faraone, Jonathan Rubin

**Affiliations:** ^1^ Supernus Pharmaceuticals, Inc., Rockville, Maryland, USA.; ^2^ Department of Psychiatry, Massachusetts General Hospital, Boston, Massachusetts, USA.; ^3^ Departments of Psychiatry and of Neuroscience and Physiology, SUNY Upstate Medical University, Syracuse, New York, USA.

**Keywords:** Viloxazine Extended-Release, SPN-812, attention-deficit/hyperactivity disorder, clinical relevance, Clinical Global Impressions Scale

## Abstract

***Objectives:*** Clinical trials in psychiatry frequently report results from lengthy, comprehensive assessments to characterize a subject emotionally, cognitively, and behaviorally before and after treatment. However, the potential treatment implications of these results and how they translate into clinical practice remain unclear. Conversely, the Clinical Global Impressions (CGI) scales are quick, intuitive assessments used to assess the functional impact of a treatment in clinically relevant terms. The objectives of the present analyses are to translate scores from comprehensive assessments of symptom severity and functional impairment into clinically meaningful CGI levels.

***Methods:*** These *post-hoc* analyses use data integrated from four pivotal Phase 3 trials in attention-deficit/hyperactivity disorder (ADHD) in children and adolescents treated with the novel nonstimulant SPN-812 (Viloxazine Extended-Release). In this study, we evaluated the ADHD Rating Scale-5 (ADHD-RS-5) and Weiss Functional Impairment Rating Scale-Parent (WFIRS-P), assessments of symptom severity and functional impairment, respectively, by linking these scales with the CGI scales at baseline and end of study.

***Results:*** For participants that improved, a one-level change on the CGI-Improvement (CGI-I) was associated with a 10–15-point change on the ADHD-RS-5, and a 0.2–0.5-point change on the WFIRS-P. On the CGI-I, ratings of much improved and very much improved were associated with a percent score decrease (i.e., improvement) of ∼55% and 80% on the ADHD-RS-5 and ∼40% and 70% on the WFIRS-P, respectively. Differences between children and adolescents were minor and are unlikely to be clinically meaningful.

***Conclusion:*** These *post-hoc* analyses provide clinically meaningful benchmarks for the interpretation of scores on the ADHD-RS-5 and WFIRS-P in terms of CGI evaluations in subjects with ADHD. These results may be useful for physicians seeking to understand a treatment's potential impact on their ADHD patients or for researchers looking to define their study results within a clinically relevant context.

Data are from clinical trials NCT03247530, NCT03247543, NCT03247517, and NCT03247556.

## Introduction

Clinical trials of psychotherapeutic drugs typically use detailed psychometric instruments to assess patients with respect to symptom severity and functional impairment. However, many treating physicians are relatively unfamiliar with these detailed, comprehensive item-by-item assessments, and routinely use instead the brief, holistic Clinical Global Impressions (CGI) scales when conducting patient evaluations (Busner and Targum 2007). The CGI scales are two separate one-item assessments used to measure either illness severity through the CGI-Severity of Illness (CGI-S) scale or change from baseline (CFB) using the CGI-Improvement (CGI-I) scale (Guy 1976). These scales can succinctly provide an overall index of patient illness, and disease-specific subscales have been validated for multiple psychiatric conditions, such as depression (Leon et al. 1993) and schizophrenia (Haro et al. 2003).

Although no attention-deficit/hyperactivity disorder (ADHD)-specific CGI subscale currently exists, as a holistic clinical assessment, the CGI generalizes well to a variety of psychiatric conditions, has been demonstrably reliable, and is suitable for routine clinical use (Berk et al. 2008). It is reported ubiquitously in clinical trials treating children and adults for a variety of disorders, including ADHD (Wilens et al. 1999, 2001; Spencer et al. 2005; Safren et al. 2010; Sprich et al. 2016; Nasser et al. 2020). On the CGI-S, patients are assessed relative to the larger patient population, using clinically relevant, qualitative terms to evaluate illness severity (e.g., not at all ill, minimally ill, severely ill).

On the CGI-I, the change in patient illness is assessed relative to their baseline condition (e.g., minimally improved, much improved, very much improved). While there is no universally agreed upon or standardized definition of what constitutes clinically meaningful change, it encompasses elements of recognizable change, normative functioning, or failure to meet diagnostic criteria (Jacobson and Truax 1991).

Limitations of the CGI for which it has been criticized are that it is too broad, inconsistent, and in the case of the CGI-I, too reliant on rater memory (Busner et al. 2009; Forkmann et al. 2011). CGI ratings can also be contaminated by separate but co-occurring conditions: patients presenting with comorbidities or adverse events can be perceived as more severely impacted by their illness than is accurate (Busner et al. 2009). These limitations can be mitigated by ensuring that the CGI is administered by a rater trained in contemporary guidelines (Busner and Targum 2007) who is familiar with the specific disorder (i.e., ADHD) and its typical progression with treatment (Guy 1976). Despite these limitations, it remains a popular tool for clinicians due to its conciseness, ease of administration, and reliability when utilized appropriately.

Across illnesses such as depression (Choi et al. 2014; Lepping et al. 2017; Leucht et al. 2018), schizophrenia (Leucht et al. 2005, 2006; Leucht and Engel 2006; Levine et al. 2008), and ADHD (Goodman et al. 2010), investigators have attempted to ascribe clinical relevance to the symptom and functional assessments used in research trials to facilitate physician interpretation of the relevance of study results to their patients. To this end, statistical score comparisons such as equipercentile linking have been used to associate scores from one assessment with scores from another, such as linking scores on the ADHD Rating Scale [ADHD-RS; based on the Diagnostic and Statistical Manual of Mental Disorders, 5th edition (DSM-5; American Psychiatric Association 2013) criteria] with the CGI scales (Goodman et al. 2010). Equipercentile linking convert scores on one scale to scores on another by linking scores with the same percentile rank, regardless of which participant generated each score (Shea and Norcini 1995; Kolen and Brennan 2014). The equipercentile link function allows for greater accuracy than many other score comparisons (e.g., mean or linear equating) as it can accurately represent curvilinear relationships (Shea and Norcini 1995; Kolen and Brennan 2014).

A previous report by Goodman et al. (2010) using an equipercentile link function analysis linked scores on the ADHD-RS, fourth edition (ADHD-RS-IV) with scores on the CGI scales, conducted on data from children and adults with ADHD treated with either placebo or the stimulant lisdexamfetamine. These authors reported that a one-level change on the CGI-I was associated with an ∼10–15-point change on the ADHD-RS-IV, and a CGI-I assessment of much improved or very much improved was associated with a minimum change on the ADHD-RS-IV of ∼50% improvement, irrespective of whether subjects were treated with placebo or lisdexamfetamine.

In this study, we build on these results by comparing scores from two comprehensive assessments separately evaluating ADHD symptom severity and functional impairment with corresponding CGI-S and CGI-I scores using equipercentile linking, conducted on pooled data from four pivotal Phase 3 clinical trials assessing the efficacy and safety of SPN-812 (Viloxazine Extended-Release) for the treatment of ADHD in children and adolescents. Using these data, the present analyses (1) report similar results to a previous analysis by Goodman et al. (2010) linking ADHD-RS-IV scores with CGI-S/CGI-I scores in children with ADHD treated with placebo or lisdexamfetamine, (2) expand on these results by linking ADHD-RS, fifth edition (ADHD-RS-5) scores with CGI scores in an adolescent ADHD population from two studies of SPN-812, and (3) present novel data linking scores on the Weiss Functional Impairment Rating Scale-Parent Version (WFIRS-P) scale with CGI-S/CGI-I in both children and adolescents with ADHD.

## Methods

### Data sources

These analyses were conducted using pooled data from four pivotal Phase 3 trials assessing the efficacy and safety of SPN-812 for the treatment of ADHD in children 6–11 years: study P301, clinicaltrials.gov NCT03247530 (Nasser et al. 2020), and study P303, NCT03247543 (Nasser et al. 2019b), and adolescents 12–17 years: study P302, NCT03247517 (Nasser et al. 2019a) and study P304, NCT03247556 (Nasser et al. 2019c) (Table 1). All four trials were randomized, double-blind, placebo-controlled, multicenter, three-arm, parallel-group studies evaluating efficacy and safety of SPN-812, a novel agent with demonstrated activity at serotonin receptors and the norepinephrine transporter (Yu et al. 2020), in pediatric patients with ADHD.

**Table 1.** Summary of Phase 3 Clinical Trials Evaluating SPN-812 in Children and Adolescents

Age group	Children (6–11 years)		Adolescents (12–17 years)	
Study number	P301	P303	P302	P304
*N* randomized/ITT population	477/460	310/301	313/301	297/292
Treatment *N*^a^ SPN-812/placebo	305/155	204/97	197/94	196/96
SPN-812 doses (per day)	100 mg, 200 mg	200 mg, 400 mg	200 mg, 400 mg	400 mg, 600 mg
Weeks (t + m)	6 (1 + 5)	8 (≤3 + 5)	6 (1 + 5)	7 (2 + 5)
End of study assessment	Week 6 (day 42)	Week 8 (day 56)	Week 6 (day 42)	Week 7 (day 49)

^a^ Based on the ITT population.

ITT, intent-to-treat; m, maintenance dosing; t, titration dosing.

In each study, symptoms of ADHD were measured according to the diagnostic criteria of the DSM-5, and the diagnosis of ADHD was confirmed with the Mini International Neuropsychiatric Interview for Children and Adolescents (MINI-KID). All participants were required to have a minimum ADHD-RS-5 total score of 28 at screening and baseline, and a minimum CGI-S score of 4 at screening. For any participant on ADHD medication before the study, drug washout was required for at least 1 week before randomization. An investigator/clinician trained in all scales administered the CGI-S at baseline only, the ADHD-RS-5 at baseline and each postbaseline study visit, and the CGI-I at each postbaseline study visit. The parent completed the WFIRS-P at the baseline and end of treatment or early termination visit (end of study [EOS]).

Exclusion criteria included a current diagnosis of any major psychiatric disorders (major depressive disorder was allowed if the subject was free of episodes at the time of screening and for 6 months prior), major neurological disorders or history of seizure disorder within the immediate family, current evidence of significant systemic disease, evidence of suicidality within 6 months, body mass index >95th percentile for age and gender, history of receiving any investigational drug within the longer of 30 days or five half-lives before day 1 dosing of SPN-812, or any other reason which might have prevented the subject from participating in the study (as determined by the investigator). Participants were required to discontinue any ADHD medications at least 1 week before baseline/randomization and to refrain from taking any ADHD medications (other than the study medication) throughout the study until EOS.

Eligible participants were randomized at baseline in a 1:1:1 ratio to either placebo or one of two doses of once-daily SPN-812 as follows: children (6–11 years of age) received either 100 or 200 mg in study P301 and either 200 or 400 mg in study P303; adolescents (12–17 years of age) received either 200 or 400 mg in study P302 or either 400 or 600 mg in study P304 (Table 1). Subjects who received active treatment took an initial dose of 100 mg (children) or 200 mg (adolescents) during week 1 and were then titrated up (if necessary) by 100 or 200 mg per week (respectively) over 1–3 weeks in a blinded fashion to their assigned target dose. Subjects maintained target, fixed dose for 5 weeks until EOS. The primary endpoint was the CFB at EOS in the ADHD-RS-5 Total score. Two key secondary endpoints were the mean CGI-I score at EOS and the CFB at EOS in the WFIRS-P Total Average score.

The study protocol was approved by the Advarra Institutional Review Board (IRB) and conducted in accordance with the Helsinki Declaration and the International Council for Harmonization Note for Guidance on Good Clinical Practice. Parents or legal guardians provided written informed consent for all study procedures, including protocol amendments. All versions of the informed consent were reviewed and approved by the IRB.

### Assessments

#### Clinical Global Impressions

The CGI scales are two single-item, stand-alone assessments of a clinician's view of a patient's overall functioning that is nonspecific to any one disease, and are thus widely used in psychiatric evaluations (Guy 1976; Busner and Targum 2007). Consisting of two companion assessments and conducted by a clinician familiar with the illness and typical treatment expectations, the CGI-S assesses a patient's global functioning at baseline relative to the larger patient population, whereas the CGI-I assesses how much a patient's illness has improved or worsened relative to their baseline state (i.e., as assessed by the CGI-S).

Both scales (CGI-S and CGI-I) are rated on a 7-point Likert scale from 1 (“normal, not at all ill,” or “very much improved,” respectively) to 7 (“extremely ill” or “very much worse,” respectively). CGI-S rankings from 1 to 7 are described as “normal, not at all ill,” “borderline mentally ill,” “mildly ill,” “moderately ill,” “markedly ill,” “severely ill,” and “among the most extremely ill.” CGI-I rankings from 1 to 7 are described as “very much improved,” “much improved,” “minimally improved,” “no change,” “minimally worse,” “much worse,” and “very much worse.”

After an initial clinical evaluation, taking into account a patient's symptoms, behavior, and circumstances, an experienced rater can complete the CGI in typically less than a minute. Across each of the four studies evaluated here, the minimum score on the CGI-S for inclusion was 4 (“moderately ill”). Successful therapy is indicated by a lower overall score in subsequent testing. In each of the four trials, the CGI-S was administered at screening and baseline, and the CGI-I was administered at each weekly, postbaseline study visit, including EOS.

#### ADHD Rating Scale-fifth edition

The ADHD-RS is an ADHD-specific rating scale designed and validated to assess current ADHD symptomatology as described in the DSM-5, currently in its fifth edition (ADHD-RS-5), and is one of the most frequently used assessments in ADHD clinical trials (Faries et al. 2001; DuPaul et al. 2016). The scale consists of 18 items that directly correspond to the 18 DSM-5 ADHD symptoms, which are further subdivided into two subscales (9 symptoms/items per subscale): Inattention and Hyperactivity/Impulsivity. On the ADHD-RS-5 scale, the individual rates the frequency of each symptom or behavior over the preceding week on a 4-point Likert scale ranging from 0 (no or rare symptoms) to 3 (severe or frequent symptoms). The sum of scores for the 18 items provides the total score (ranging between 0 and 54).

In the four Phase 3 trials, a trained investigator/clinician administered and scored the ADHD-RS-5 Home Version Child (P301/P303) or Adolescent (P302/P304) instrument at screening, baseline, and at each weekly postbaseline study visit through to EOS. ADHD-RS-5 Total scores were used in the present analyses.

#### WFIRS-Parent

The WFIRS-P assesses functional impairment in children and adolescents by quantifying the degree to which a patient's ADHD-related symptoms affect daily activities (e.g., school tasks, relationships) over the past month (Gajria et al. 2015). The parent rates the impact of their child's emotional or behavioral problems across six domains: family, school and learning, life skills, child's self-concept, social activities, and risky activities. Each of the 50 items is rated on a 4-point Likert scale from 0 (never or not at all) to 3 (very often or very much) (or “not applicable” if not relevant). The WFIRS-P results in an overall Total Average score and an average score for each of the six subdomains, where higher scores are associated with higher degrees of functional impairment. For all four trials, the WFIRS-P was administered at baseline and at EOS, and the Total Average score was used in the present analyses.

### Statistical analyses

Equipercentile linking was used to link scores on the ADHD-RS-5 and WFIRS-P with corresponding scores on the CGI (Shea and Norcini 1995; Kolen and Brennan 2014). This technique identifies scores on both measures that have the same percentile rank, and has been used extensively in research on schizophrenia (Leucht et al. 2005, 2006; Leucht and Engel 2006; Levine et al. 2008), depression (Choi et al. 2014; Lepping et al. 2017; Leucht et al. 2018), ADHD (Goodman et al. 2010), pain (Cook et al. 2015), and cancer (Namiki et al. 2007) to translate between different assessments. Equipercentile linking identifies scores on two measures (e.g., ADHD-RS-5 and the CGI-S) that have the same percentile rank, regardless of linearity, size or shape of the distributions, or which subject produced each score.

In this study, we created link functions for two time points (baseline and EOS) in each of the four trials to (1) convert scores on each of the four scales (CGI-S, CGI-I, ADHD-RS-5, and WFIRS-P) to percentile ranks using a percentile rank function, and (2) match scores on each scale that have the same percentile rank, and plot them as X, Y pair values. From this, a link function is generated for each matched scale, and, by using this function, scores on one scale can be translated into the other scale. This method links scores with the same percentile rank, regardless of which participant generated those scores, therefore, individual participant scores are not considered in the equipercentile linking analysis.

Although scores on our scales are discrete, the equipercentile link function is continuous, thus, each score is expanded to encompass a range, for example, a CGI-S score of 4 (moderately ill) is represented here by any score 3.5–4.4, a score of 5 (markedly ill) is represented by 4.5–5.4, a score of 6 (severely ill) is represented by 5.5–6.4, and a score of 7 (extremely ill) is represented by 6.5–7. All scores are presumed to be uniformly distributed within the defined range, for example, all scores of 5 on the CGI-S are presumed to be uniformly distributed within the predefined 4.5–5.4 range. For each age group's link function, 95% confidence intervals (CIs) were calculated using a bootstrap imputation method, where data were imputed 200 times with replacement (i.e., CIs were generated for children and adolescents separately, collapsed across individual study).

Analyses were conducted on the intent-to-treat populations of all four trials, defined as all subjects randomized to treatment with both a baseline score and at least one postrandomization score on the CGI-I and at least one other assessment. The four clinical trials used as input to our analyses required minimum baseline scores of 28 on the ADHD-RS-5 and 4 on the CGI-S, with upward resulting ranges of 54 and 7, respectively. Eight subjects were inadvertently randomized despite having ADHD-RS-5 scores below 28: one child (score: 23) and seven adolescents (range of scores: 11–27). Thus, our link functions were based on data input from these ranges, and include subjects treated from 1 to 8 weeks, provided they had a baseline score and at least one postrandomization score as defined above.

The endpoint of each assessment was defined as the last postrandomization treatment week for which a valid score was obtained (i.e., EOS), and only subjects with both baseline and EOS scores were included in each analysis. Across both assessments, the analysis was conducted on baseline scores, absolute CFB scores at EOS, and percent CFB scores at EOS.

Analyses of baseline scores and treatment effects assessed by the CGI-I were conducted in GraphPad Prism (version 8.4.3, San Diego, CA). Link function analyses were conducted in SAS (version 9.4, Cary, NC). To compare results from the present analyses with previously published analyses linking ADHD-RS-IV and CGI scores in children with ADHD treated with either placebo or lisdexamfetamine, we used Origin Pro (OriginLab Corporation, Northampton, MA) to extract data from the equipercentile link functions published in Goodman et al. (2010). We plotted the resulting functions with those generated in the present analyses after treatment with SPN-812 for comparison between lisdexamfetamine (a stimulant prodrug) and SPN-812 (a nonstimulant) trials.

## Results

### Demographics, baseline characteristics, and treatment effects

#### Demographics

The study groups were balanced with regard to demographic characteristics. For children, the mean age was 8.5 years (standard deviation [SD] = 1.69). The majority of participants were male (63.8%), and 36.3% were female. Most participants were White (52.05%); 42.6% were Black or African American, 0.7% were American Indian or Alaska Native, 0.3% were Asian, and 4.3% were of multiple races. The majority of participants were Not Hispanic or Latino (72.0%), whereas 27.9% were Hispanic or Latino, and 0.1% were of unknown ethnicity. The mean height was 134.46 cm (SD = 11.30) and the mean weight was 31.63 kg (SD = 8.40).

For adolescents, the mean age was 13.9 years (SD = 1.58). The majority of participants were male (65.6%), and 34.4% were female. Participants were predominantly White (61.4%); 34.2% were Black or African American, 0.8% were American Indian or Alaska Native, 0.3% were Asian, 0.2% were Native Hawaiian or Other Pacific Islander, and 3.0% were of multiple races. The majority of participants were Not Hispanic or Latino (68.5%), whereas 31.4% were Hispanic or Latino, and 0.2% were of unknown ethnicity. The mean height was 163.26 cm (SD = 10.36) and the mean weight was 57.27 kg (SD = 13.02).

#### Baseline characteristics

To identify group differences at baseline, we performed three separate three-way analyses of variance of ADHD-RS-5, WFIRS-P, and CGI-S scores analyzing the effects of age (child vs. adolescents), study (first vs. second study), and assigned treatment group (placebo vs. SPN-812) on baseline scores. Each of the three analyses revealed a significant main effect of age (all *p*-values <0.0001), no effect of study (all *p*-values >0.05), no effect of assigned treatment group (all *p*-values >0.05), and no two- or three-way interactions between these factors (all *p*-values >0.05).

On all three measures, children were evaluated at baseline as having higher scores (i.e., greater illness) than adolescents, regardless of study or assigned treatment group (average baseline ADHD-RS-5 scores for children = 44.2, adolescents = 39.9; average baseline WFIRS-P for children = 1.11, adolescents = 0.99; average baseline CGI-S for children = 4.81, adolescents = 4.62, thus markedly ill). However, this effect is not likely to be clinically meaningful across each measure.

#### Treatment effects

**FIG. 1.**
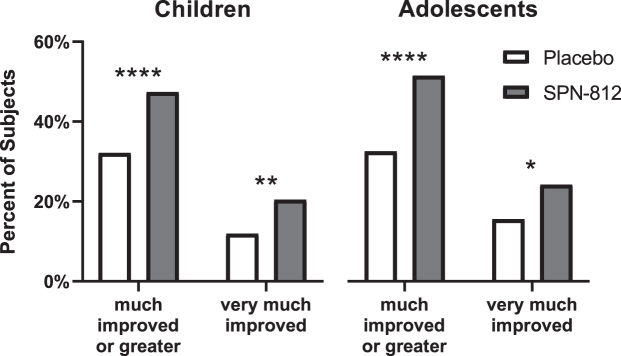
Treatment effects as assessed by the CGI-I. More subjects treated with SPN-812 achieved clinically meaningful improvements (i.e., CGI-I assessment of much improved or very much improved) than subjects receiving placebo. Fisher's exact test, two-sided. *p < 0.05, **p < 0.01, ****p < 0.0001. CGI, Clinical Global Impressions; CGI-I, CGI-Improvement.

A two-sided Fisher's exact test compared the number of subjects treated with SPN-812 versus placebo achieving clinically meaningful improvements at EOS (i.e., an evaluation of much improved or greater on the CGI-I at EOS) (Fig. 1). Significantly more children treated with SPN-812 (47.35%) achieved a CGI-I evaluation of much improved or very much improved (i.e., CGI-I score = 2 or 1) compared with children treated with placebo (32.14%) (*p* < 0.0001). Similarly, more children treated with SPN-812 (20.43%) were evaluated at EOS as very much improved (i.e., a score of 1) compared with children treated with placebo (11.90%) (*p* < 0.01).

Among adolescents, significantly more participants treated with SPN-812 (51.40%) were evaluated as much improved or very much improved at EOS compared with adolescents treated with placebo (32.50%) (*p* < 0.0001). Similarly, more adolescents treated with SPN-812 (24.17%) were evaluated as very much improved at EOS relative to adolescents treated with placebo (15.50%) (*p* < 0.05).

### Linking ADHD-RS-5 scores with the CGI

#### Baseline ADHD-RS-5 total scores and CGI-S levels

**FIG. 2.**
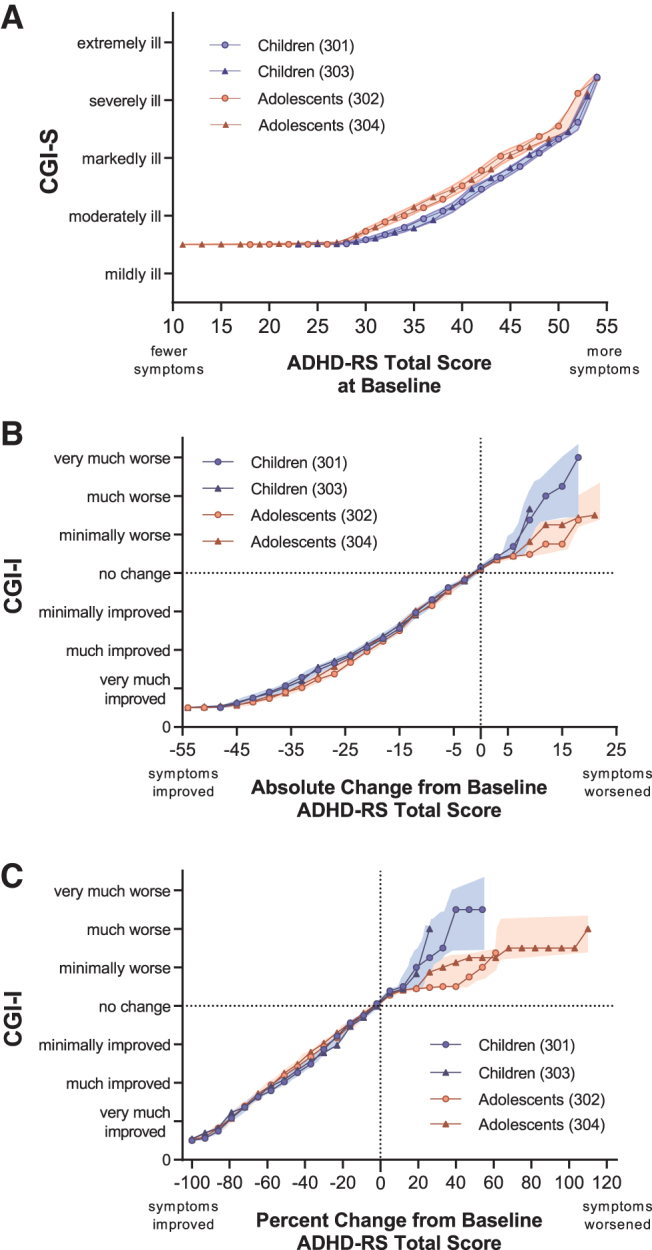
Link functions for ADHD-RS scores and CGI-S/CGI-I levels. Children (blue lines) and adolescents (red lines) are from the present analysis in pediatric patients treated with SPN-812 or placebo. Shaded bands represent 95% confidence intervals for link functions by age group, that is, collapsed across study. For clarity, some points were omitted without changing the shape of the line: included on **(A)** and **(B)** is every second point; on **(C)**, every sixth point. **(A)** Baseline ADHD-RS-5 Total scores and CGI-S levels. **(B)** ADHD-RS-5 Total score absolute change from baseline and CGI-I levels at the end of study. **(C)** ADHD-RS-5 Total score percent change from baseline and CGI-I levels at the end of study. ADHD-RS-5, Attention-Deficit/Hyperactivity Disorder Rating Scale 5; CGI, Clinical Global Impressions; CGI-I, CGI-Improvement; CGI-S, CGI-Severity.

The equipercentile link functions for baseline ADHD-RS-5 and CGI-S levels in children and adolescents are shown in Figure 2A. The link functions matching baseline ADHD-RS-5 scores to CGI-S scores differed between children and adolescents (as indicated by lack of overlap of 95% CIs at values between moderately and markedly ill). In children, an ADHD-RS-5 score (median [range]) of 37 (23–41) was linked with the CGI-S level of moderately ill, 47 (42–51) linked with markedly ill, and 53 (52–54) with severely or extremely ill.

In adolescents, an ADHD-RS-5 score of 34 (11–39) linked with moderately ill, 44 (40–50) linked with markedly ill, and 52 (51–54) linked with severely or extremely ill. CGI-S levels corresponding with total ADHD-RS-5 scores based on this link function are described in Table 2. Summary statistics, including quartiles and ranges used to generate the link function are shown in Supplementary Table S1, and a figure illustrating this link function by treatment group is shown in the Supplementary Figure S1.

**Table 2.** Attention-Deficit/Hyperactivity Disorder Rating Scale 5 Values Corresponding to Clinical Global Impressions Levels Derived from the Link Function

Patient population	CGI-S/CGI-I	*N*	Midpoint	Range
Baseline				
Overall	4—Moderately ill	556	35	11–40
	5—Markedly ill	626	46	41–51
	6—Severely ill	162	53	52–54
	7—Extremely ill	10		
Children	4—Moderately ill	266	37	23–41
	5—Markedly ill	384	47	42–51
	6—Severely ill	103	53	52–54
	7—Extremely ill	8		
Adolescents	4—Moderately ill	290	34	11–39
	5—Markedly ill	242	44	40–50
	6—Severely ill	59	52	51–54
	7—Extremely ill	2		
Absolute change from baseline				
Overall	1—Very much improved	260	−36	−29 to −54
	2—Much improved	329	−22	−16 to −28
	3—Minimally improved	301	−12	−7 to −15
Children	1—Very much improved	134	−37	−31 to −54
	2—Much improved	188	−22	−16 to −30
	3—Minimally improved	164	−11	−7 to −15
Adolescents	1—Very much improved	126	−34	−27 to −52
	2—Much improved	141	−21	−16 to −26
	3—Minimally improved	137	−11	−7 to −15
Percent change from baseline				
Overall	1—Very much improved	260	−81	−70 to −100
	2—Much improved	329	−55	−41 to −69
	3—Minimally improved	301	−29	−17 to −40
Children	1—Very much improved	134	−82	−70 to −100
	2—Much improved	188	−54	−38 to −69
	3—Minimally improved	164	−27	−17 to −37
Adolescents	1—Very much improved	126	−80	−70 to −100
	2—Much improved	141	−56	−44 to −69
	3—Minimally improved	137	−31	−17 to −43

CGI, Clinical Global Impressions; CGI-I, CGI-Improvement; CGI-S, CGI-Severity.

#### Absolute CFB ADHD-RS-5 scores and CGI-I levels at EOS

The equipercentile link functions for absolute CFB ADHD-RS-5 and CGI-I levels in children and adolescents are shown in Figure 2B. The link functions matching absolute CFB ADHD-RS-5 scores to CGI-I scores did not appear to differ between children and adolescents. In children, an absolute CFB in ADHD-RS-5 scores (median [range]) of −37 (−31 to −54) was linked with the CGI-I rating of very much improved, −22 (−16 to −30) linked with much improved, and −11 (−7 to −15) with minimally improved. In adolescents, a change in ADHD-RS-5 scores of −34 (−27 to −52) was linked with the CGI-I rating of very much improved, −21 (−16 to −26) linked with much improved, and −11 (−7 to −15) with minimally improved.

When considering the conventional measure for meaningful clinical improvement (much improved or very much improved combined), the median (range) of absolute CFB for children was linked with −30 (−16 to −54), and −26 (−16 to −52) for adolescents. CGI-I levels corresponding with the absolute CFB ADHD-RS-5 scores based on this link function are described in Table 2. Summary statistics used to generate the link function are shown in Supplementary Table S2, and a figure illustrating this link function by treatment group is shown in the Supplementary Figure S1.

#### Percent CFB ADHD-RS-5 scores and CGI-I levels at EOS

The equipercentile link functions for percent CFB ADHD-RS-5 scores and CGI-I levels in children and adolescents are shown in Figure 2C. In children, the percent CFB in ADHD-RS-5 scores (median [range]) of −82 (−70 to −100) was linked with the CGI-I rating of very much improved, −54 (−38 to −69) linked with much improved, and −27 (−17 to −37) with minimally improved. In adolescents, a percent change in ADHD-RS-5 scores of −80 (−70 to −100) was linked with the CGI-I rating of very much improved, −56 (−44 to −69) linked with much improved, and −31 (−17 to −43) with minimally improved.

When considering the conventional measure for meaningful clinical improvement (much improved or very much improved combined), the median (range) of absolute CFB for children was linked with −70 (−38 to −100), and −69 (−44 to −100) for adolescents. CGI-I levels corresponding with the percent CFB ADHD-RS-5 scores based on this link function are described in Table 2. Summary statistics used to generate the link function are shown in Supplementary Table S3, and a figure illustrating this link function by treatment group is shown in the Supplementary Figure S1.

#### Replicability of equipercentile linking of ADHD-RS scores with CGI levels

**FIG. 3.**
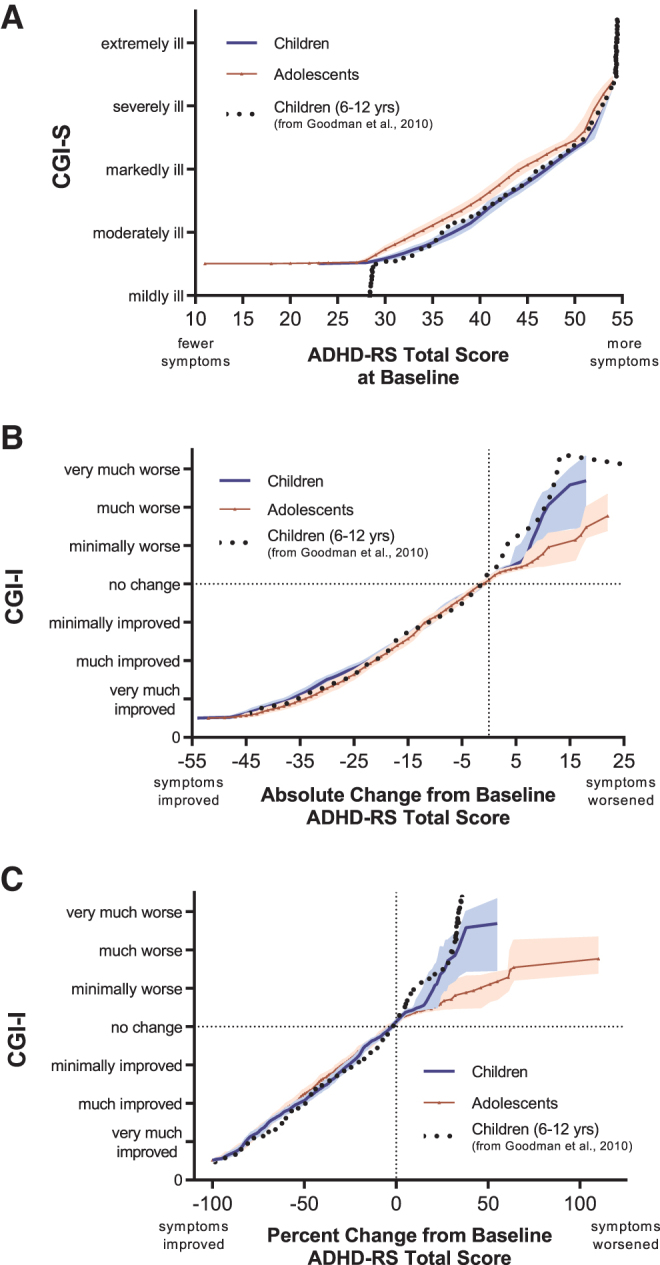
Link functions for ADHD-RS scores and CGI-S/CGI-I Levels compared with a previous trial of lisdexamfetamine. children (blue line) and adolescents (red triangles) are from the present analysis in pediatric patients treated with SPN-812 or placebo (using ADHD-RS-5); Children 6–12 years (black dotted line) are from a previously-published analysis in children treated with lisdexamfetamine or placebo using ADHD-RS-IV (Goodman et al. 2010). Shaded bands represent 95% confidence intervals by age group. For clarity, some points were omitted without changing the shape of the line: included on **(A)** and **(B)** is every second point; on **(C)**, every sixth point. **(A)** Baseline ADHD-RS Total scores and CGI-S levels. **(B)** ADHD-RS Total score absolute change from baseline and CGI-I levels at the end of study. **(C)** ADHD-RS Total score percent change from baseline and CGI-I levels at the end of study. ADHD-RS, Attention-Deficit/Hyperactivity Disorder Rating Scale; CGI, Clinical Global Impressions; CGI-I, CGI-Improvement; CGI-S, CGI-Severity.

To evaluate the reliability of these analyses, we overlaid the link functions generated in this study with those previously reported in children with ADHD treated with lisdexamfetamine or placebo linking the CGI scales with the ADHD-RS-IV (Goodman et al. 2010). Link functions were extracted for scores at baseline (black dotted line in Fig. 3A), the absolute CFB at EOS (Fig. 3B), and the percent CFB at EOS (Fig. 3C). There was no appreciable difference between children in the lisdexamfetamine study (6–12 years) and the SPN-812 studies (6–11 years) at baseline, or on functions linking absolute or percent CFB scores, as evidence by the lisdexamfetamine function being relatively contained within the 95% CIs of the children from the SPN-812 studies.

### Linking WFIRS-P scores with the CGI

#### Baseline WFIRS-P scores and CGI-S levels

**FIG. 4.**
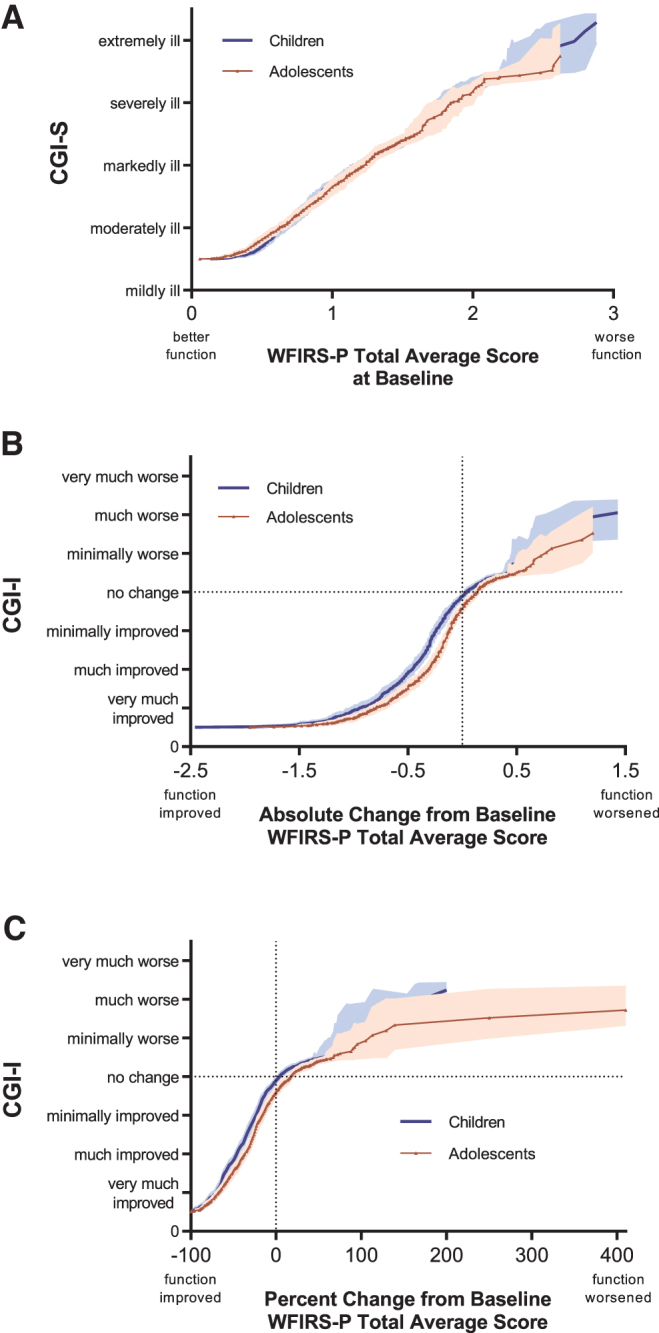
Link functions for WFIRS-P scores and CGI-S/CGI-I Levels. Children (blue line) and adolescents (red triangles) are from the present analysis in pediatric patients treated with SPN-812 or placebo. Shaded bands represent 95% confidence intervals by age group. **(A)** Baseline WFIRS-P Total Average scores and CGI-S levels. **(B)** WFIRS-P Total Average scores absolute change from baseline and CGI-I levels at the end of study. **(C)** WFIRS-P Total Average scores percent change from baseline and CGI-I levels at the end of study. CGI, Clinical Global Impressions; CGI-I, CGI-Improvement; CGI-S, CGI-Severity; WFIRS-P, Weiss Functional Impairment Rating Scale-Parent.

The equipercentile link functions for baseline WFIRS-P scores and CGI-S levels in children and adolescents are shown in Figure 4A. The link functions matching baseline WFIRS-P scores to CGI-S scores did not significantly differ between children and adolescents. In children, a WFIRS-P score (median [range]) of 0.65 (0.14–0.88) was linked with the CGI-S level of moderately ill, 1.20 (0.89–1.61) linked with markedly ill, 1.78 (1.62–2.29) with severely ill, and 2.54 (2.36–2.88) with extremely ill.

In adolescents, a WFIRS-P score of 0.62 (0.06–0.92) linked with moderately ill, 1.22 (0.94–1.62) linked with markedly ill, 1.83 (1.64–2.57) linked with severely ill, and 2.60 (2.57–2.86) linked with extremely ill. CGI-S levels corresponding with the WFIRS-P scores based on this link function are described in Table 3. Summary statistics, including quartiles and ranges used to generate the link function, are shown in Supplementary Table S4, and a figure illustrating this link function by treatment group is shown in the Supplementary Figure S2.

**Table 3.** Weiss Functional Impairment Rating Scale-Parent Values Corresponding to Clinical Global Impression Levels Derived from the Link Function

Patient population	CGI-S/CGI-I	*N*	Midpoint	Range
Baseline				
Overall	4—Moderately ill	522	0.64	0.06–0.90
	5—Markedly ill	571	1.21	0.90–1.62
	6—Severely ill	149	1.80	1.63–2.45
	7—Extremely ill	10	2.57	2.46–2.88
Children	4—Moderately ill	247	0.65	0.14–0.88
	5—Markedly ill	351	1.20	0.89–1.61
	6—Severely ill	94	1.78	1.62–2.29
	7—Extremely ill	8	2.54	2.36–2.88
Adolescents	4—Moderately ill	275	0.62	0.06–0.92
	5—Markedly ill	220	1.22	0.94–1.62
	6—Severely ill	55	1.83	1.64–2.57
	7—Extremely ill	2	2.60	2.57–2.86
Absolute change from baseline				
Overall	1—Very much improved	255	−0.86	−0.60 to −2.46
	2—Much improved	303	−0.40	−0.28 to −0.60
	3—Minimally improved	276	−0.18	−0.08 to −0.28
Children	1—Very much improved	130	−0.94	−0.66 to −2.46
	2—Much improved	169	−0.46	−0.32 to −0.65
	3—Minimally improved	152	−0.24	−0.12 to −0.32
Adolescents	1—Very much improved	125	−0.81	−0.52 to −1.95
	2—Much improved	134	−0.32	−0.20 to −0.52
	3—Minimally improved	124	−0.12	−0.04 to −0.19
Percent change from baseline				
Overall	1—Very much improved	255	−69	−58 to −100
	2—Much improved	303	−43	−31 to −57
	3—Minimally improved	276	−21	−11 to −30
Children	1—Very much improved	130	−70	−61 to −100
	2—Much improved	169	−46	−35 to −60
	3—Minimally improved	152	−24	−15 to −34
Adolescents	1—Very much improved	125	−68	−52 to −100
	2—Much improved	134	−37	−27 to −51
	3—Minimally improved	124	−17	−5 to −26

CGI, Clinical Global Impressions; CGI-I, CGI-Improvement; CGI-S, CGI-Severity.

#### Absolute CFB WFIRS-P scores and CGI-I levels at EOS

The equipercentile link functions for absolute CFB WFIRS-P scores and CGI-I levels in children and adolescents are shown in Figure 4B. The link functions matching absolute CFB WFIRS-P scores to CGI-I scores appeared to differ between children and adolescents (as indicate by the nonoverlapping CIs). In children, an absolute CFB in WFIRS-P scores (median [range]) of −0.94 (−0.66 to −2.46) was linked with the CGI-I rating of very much improved, −0.46 (−0.32 to −0.65) linked with much improved, and −0.24 (−0.12 to −0.32) with minimally improved. In adolescents, a change in WFIRS-P scores of −0.81 (−0.52 to −1.95) was linked with the CGI-I rating of very much improved, −0.32 (−0.20 to −0.52) linked with much improved, and −0.12 (−0.04 to −0.19) with minimally improved.

When considering the conventional measure for meaningful clinical improvement (much improved or very much improved combined), the median (range) of absolute CFB for children was linked with −0.66 (−0.32 to −2.46), and −0.52 (−0.20 to −1.95) for adolescents. CGI-I levels corresponding with the WFIRS-P scores based on this link function are described in Table 3. Summary statistics used to generate the link function are shown in Supplementary Table S5, and a figure illustrating this link function by treatment group is shown in the Supplementary Figure S2.

#### Percent CFB WFIRS-P scores and CGI-I levels at EOS

The equipercentile link functions for percent CFB WFIRS-P scores and CGI-I levels in children and adolescents are shown in Figure 4C. In children, the percent CFB in WFIRS-P scores (median [range]) of −70 (−61 to −100) was linked with the CGI-I rating of very much improved, −46 (−35 to −60) linked with much improved, and −24 (−15 to −34) with minimally improved. In adolescents, a percent change in WFIRS-P scores of −68 (−52 to −100) was linked with the CGI-I rating of very much improved, −37 (−27 to −51) linked with much improved, and −17 (−5 to −26) with minimally improved.

When considering the conventional measure for meaningful clinical improvement (much improved or very much improved combined), the median (range) of absolute CFB for children was linked with −61 (−35 to −100), and −52 (−27 to −100) for adolescents. CGI-I levels corresponding with the WFIRS-P scores based on this link function are described in Table 3. Summary statistics, including quartiles and ranges used to generate the link function, are shown in Supplementary Table S6, and a figure illustrating this link function by treatment group is shown in the Supplementary Figure S1.

## Discussion

The current analyses link scores on psychometrically validated assessments commonly used in clinical trials for ADHD with the clinician-preferred, clinically relevant CGI scales using data from four identical Phase 3 studies in children and adolescents with ADHD treated with either placebo or SPN-812. The assessments used in these *post-hoc* analyses are commonly used in clinical trials of ADHD to assess symptom severity (through the ADHD-RS-5) and functional impairment (through the WFIRS-P), two related yet separate constructs. The results presented here provide practical benchmarks for translating these scores into clinically meaningful benchmarks. These results should be useful for physicians seeking to understand a treatment's potential impact on their patients or for researchers looking to define the clinical relevance of their findings.

### Quantifying clinically meaningful change

While there is no standard definition for what constitutes clinically meaningful change after therapy, Jacobson and Truax (1991) propose a twofold model in which the definition of clinically meaningful encompasses (1) a recognizable change in condition, and (2) a statistically quantifiable level of functioning closer to that of a normative population or a failure to meet the diagnostic criteria for the disease. Quantifying recognizable change has also been done through analysis of the minimally important difference (MID) (or minimal clinically important difference [MCID]), typically defined as the change in score associated with a patient's recognition of improvement (Zhang et al. 2005; Hodgkins et al. 2017). When applied to the ADHD-RS scale (fourth edition), a previous report determined the MCID to be a 10.2-point change in the total score, or a 27% decrease from baseline (Zhang et al. 2005).

These standards align well with the present analyses, where we report a one-level change in CGI-I scores between the ranges of “no change” to “very much improved” to be associated with an absolute change in ADHD-RS-5 total scores of 10–15 points, or a percent change of 25%–30% (Table 2), a range commonly used in clinical trials to identify treatment “responders” (Spencer et al. 2001; Michelson et al. 2002; Kelsey et al. 2004; Kemner et al. 2005).

A separate report identified the WFIRS-P MID to be a 0.25 decrease in the total mean score (Hodgkins et al. 2017; Weiss et al. 2018), again consistent with our results where a one-level CGI-I change from “no change” to “minimally improved” to “much improved” was associated with an absolute change in median WFIRS-P total average scores of 0.2–0.28 (Table 3). Unexpectedly, in our data, the change from “much improved” to “very much improved” was larger—approximately 0.48—for both children and adolescents. This nonlinearity validates the use of equipercentile linking for associating scores on different assessments, as equipercentile linking can accurately represent curvilinear relationships (Shea and Norcini 1995; Kolen and Brennan 2014).

Although there are no standardized descriptions anchoring the 7 points of the CGI scales, some researchers have proposed that a one-level change on the CGI-S is considered a recognizable change in illness, and thus the MID for this scale (Zhang et al. 2005). However, the goal of treatment should be not just a recognizable change in condition, but a clinically relevant change that indicates either a significant improvement or normalization of illness/illness remission, that is, a patient initially evaluated as extremely ill before treatment and severely ill after treatment may have experienced a measurable change in condition, but likely not a sufficiently large improvement to justify continuing the same therapy.

More detailed guidelines describing each CGI-I level in terms of symptoms, functional impairment, or appropriate clinical action (e.g., at what score should medication be changed, etc.) associate “minimally improved” with no clinically meaningful reduction of symptoms and very little change in functioning, whereas the CGI-I level “much improved” is described in terms of a significant reduction of symptoms and increase in functioning (Kay 1990; Busner and Targum 2007). By these criteria, a CGI-I assessment of “much improved” would be the minimum change in illness indicative of clinically meaningful improvement.

The results described in this study and in a prior report (Goodman et al. 2010) can be used to assess the clinical impact of results from clinical trials by translating scores on the ADHD-RS into CGI levels. Taking as an example the first Phase 3 study of SPN-812 used in this analysis, P301 (Nasser et al. 2020), scores on the ADHD-RS-5 began to rapidly improve in subjects treated with SPN-812 within 1 week of treatment (absolute CFB SPN-812 = 9 points, placebo = 6 points). Although this group difference is statistically significant (*p* < 0.05), according to the present analyses an absolute CFB of 15 or fewer points is associated with minimal improvement on the CGI-I (Table 2), and as a result is unlikely to represent a satisfactory response to therapy. Notably, by week 3, the absolute CFB in SPN-812-treated subjects improved by 16 points, and further improving to over 18 points by week 5 (vs. 10 and 12 points, respectively, for subjects receiving placebo), falling within the range of much improved, and therefore likely to be recognized by patients and/or their treating physicians as clinically meaningful improvement.

### Clinical relevance of response thresholds

In the present analyses, the CGI-I level of much improved, generally understood to be the minimum level associated with clinically meaningful improvement, was associated with a score reduction from baseline of ∼55% on the ADHD-RS-5 (Fig. 2C), and 43% on the WFIRS-P (Fig. 4C). This stands in contrast to many studies where patients meeting a 25%–30% reduction in baseline symptom severity, sometimes thought to represent a CGI-I assessment of much improved (Buitelaar et al. 2003), are frequently categorized as having responded to a given treatment (Spencer et al. 2001; Michelson et al. 2002; Kelsey et al. 2004; Kemner et al. 2005). The present analysis (Fig. 2C) and a previous report (Goodman et al. 2010) suggest that a 30% reduction in baseline symptom scores is in fact associated with a minimal improvement on the CGI-I in children and adolescents (Fig. 3C).

Taken together, these data suggest that the commonly used threshold of 30%, or the less frequently used, more stringent 40% threshold (Newcorn et al. 2009; Cutler et al. 2014), may not be adequately rigorous when assessing clinically meaningful improvement and drug efficacy. Future researchers interested in applying clinically meaningful benchmarks to their studies may consider selecting a sufficiently large threshold for improvement to warrant continued treatment; that is, a response reduction closer to 55% on the ADHD-RS-5 would be required to achieve a CGI-I evaluation of much improved, and closer to 80% to achieve an evaluation of very much improved. Notably, a 50%–65% improvement on the ADHD-RS-IV was associated with clinically significant improvement on a measure of functional impairment (Buitelaar et al. 2009), further validating 50% improvement on the ADHD-RS as a meaningful response criterion.

Many researchers also assess clinically meaningful change through symptom normalization or remission, consistent with the twofold model proposed by Jacobson and Truax (1991), frequently using an ADHD-RS total score of ≤18 as a threshold for remission (Steele et al. 2006; Weiss et al. 2018, 2019). The data in the present analyses were preselected for ADHD-RS-5 scores ≥28, therefore a normative comparison with the CGI-S for these data cannot be made.

However, our data from the WFIRS-P show that a median WFIRS-P score of 0.64 is associated with the CGI-S level of moderately ill, defined in part by symptoms causing functional impairment that may warrant medication (Table 3) (Kay 1990; Busner and Targum 2007). This score is also the score on the WFIRS-P that had been previously shown to accurately discriminate pediatric patients with and without ADHD (Thompson et al. 2017). This suggests that a moderately ill CGI-S evaluation is likely to be associated with clinically meaningful functional impairment, and suggests that a WFIRS-P score of ≤0.65 is a valid threshold for defining functional remission.

### Children versus adolescents

Across all six pairs of linkages, the relationship between the comprehensive assessments and CGI levels were generally consistent between children and adolescents, as measured by 95% CIs, with the exception of baseline ADHD-RS-5 scores (Fig. 2A) and WFIRS-P CFB scores (Fig. 4B, C). At baseline, children tended to be assessed on symptom severity as more ill on the CGI-S relative to adolescents with the same ADHD-RS-5 score, a difference that became imperceptible at EOS. This was not the case when evaluating functional impairment: CGI-S and WFIRS-P link functions at baseline were similar between children and adolescents, despite having different baseline scores on the WFIRS-P (i.e., children were consistently rated as more functionally impaired than adolescents). At EOS, children were evaluated as more improved on the WFIRS-P for each CGI-I level (Figs. 4B, C), relative to adolescents.

Although the nonoverlapping CIs suggest these age differences are significant statistically, these differences are minor and are unlikely to be clinically meaningful. For instance, a CGI-S assessment of “markedly ill” was associated with an ADHD-RS-5 value of 47 in children, and 44 in adolescents (Table 2), a difference which equals 5% of the range of possible scores (1–54).

While the cause of these minor differences between child and adolescent link functions is unclear, it may reflect the nature of the assessments and changes in the clinical presentation of ADHD throughout development, which is well known to change across the lifespan (Wilens et al. 2002). Symptoms of hyperactivity and impulsivity are common in younger children and fade with development and the maturation of white matter microstructure (Francx et al. 2015), whereas subtler symptoms of inattention tend to persist into later childhood and adolescence (Biederman et al. 2000; Franke et al. 2018).

The intuitive, impressionistic nature of the CGI might be more easily influenced by these easily observed, externalized symptoms of hyperactivity and impulsivity, providing for an impression of greater illness in children and opportunity to assess greater improvement in functional behavior, versus the more covert nature of inattention that is characteristic of ADHD in adolescence (Biederman et al. 2000; Franke et al. 2018).

Whether the results reported in this study will extend to an adult population remain to be tested, although data from a trial of lisdexamfetamine (Goodman et al. 2010) suggest they are likely to be similar to those reported here in children and adolescents. A similar Phase 3 clinical trial evaluating the efficacy of SPN-812 in adults 18–65 years has recently been completed (NCT04016779).

### Limitations

Our results should be interpreted in the context of some limitations. These are *post-hoc* analyses and were not prespecified for these trials. When assessing percent CFB, because few participants experienced extreme levels of worsening, associations between scales in this region cannot be reliably interpreted, as indicated by the much wider CIs in the corresponding sections of Figures 2–4. Our inclusion criteria required an ADHD-RS-5 score of ≥28 and a CGI-S score of ≥4, further limiting the reliability in these ranges. Use of these criteria likely explains the abrupt changes in slopes in the bottom left quadrants of the figures.

These data are also from children and adolescents diagnosed with ADHD and do not include any healthy subjects, therefore precluding any conclusions about linking assessments within the normative ranges at baseline. While these analyses provide benchmarks for a evaluating a response to treatment relative to baseline per the CGI-I, they do not provide such benchmarks for the CGI-S at endpoint, thus precluding any conclusions about which ADHD-RS-5 or WFIRS-P scores are associated with remission after treatment. Although an ADHD-RS-5 score ≤18 and a WFIRS-P score of ≤0.65 are commonly used as thresholds of symptomatic and functional remission, respectively (Cutler et al. 2014; Weiss et al. 2018), whether these scores are associated with a CGI-S evaluation of 1 (normal, not at all ill) using these data was not evaluated.

Finally, the utility of these results depends on a shared understanding of which CGI-I benchmarks and descriptors constitute clinically meaningful change (to this end, a comprehensive framework is described in Busner and Targum 2007). In the absence of such a consensus, thresholds for improvement (or a lack thereof) can be somewhat arbitrary. Regardless of how these assessments may be used in the treatment of ADHD, clinicians should strive to consider the clinical impact on patients beyond simply quantifying the degree of change in a patient's condition or their baseline severity.

## Conclusions

Clinical studies of ADHD typically use ratings of symptoms and impairments to evaluate participants. These measures may be unfamiliar to physicians or impractical for them to use during regular clinical treatment. Clinicians seeking to understand how much improvement scores on the ADHD-RS-5 or WFIRS-P are associated with clinically relevant treatment outcomes can use the present analyses as guidelines to inform treatment decisions. These analyses in children, and those of a previous report (Goodman et al. 2010), and adolescents with ADHD show that a CGI-I “minimally improved” evaluation is associated with ∼30% and 20% improvement on the ADHD-RS-5 and WFIRS-P, respectively, while a CGI-I “much improved” evaluation is associated with ∼55% and 40% improvement on the ADHD-RS-5 and WFIRS-P, respectively. These analyses help place baseline and post-treatment changes in ADHD-RS-5 and WFIRS-P scores in a clinical context, provide practical benchmarks for the interpretation of these scores, and may inform future understanding of the clinical relevance of these scales.

## Supplementary Material

Supplemental data

Supplemental data

Supplemental data

Supplemental data

Supplemental data

Supplemental data

Supplemental data

Supplemental data

## Clinical Significance

Clinical trials in psychiatry frequently report results from lengthy, comprehensive assessments. However, the potential treatment implications of these results and how they translate into clinical practice remain unclear. Conversely, the CGI-I scale is a brief (i.e., 2 minutes), intuitive scale commonly used to assess the clinical impact of a given treatment, using ratings of much improved or very much improved (i.e., CGI-I scores of 1 or 2), understood to represent a clinically meaningful improvement in a patient's condition. The present *post-hoc* analyses provide clinically meaningful benchmarks for the interpretation of scores on the ADHD-RS-5 and WFIRS-P, comprehensive measures of ADHD symptoms and functional impairment, respectively, into clinically meaningful CGI levels. These results may be useful for physicians seeking to understand a treatment's potential impact on their ADHD patients or for researchers looking to define their study results within a clinically relevant context.

## References

[B1] Berk M, Ng F, Dodd S, Callaly T, Campbell S, Bernardo M, Trauer T: The validity of the CGI severity and improvement scales as measures of clinical effectiveness suitable for routine clinical use. J Eval Clin Pract 14:979–983, 2008 18462279 10.1111/j.1365-2753.2007.00921.x

[B2] Biederman J, Mick E, Faraone SV: Age-dependent decline of symptoms of attention deficit hyperactivity disorder: Impact of remission definition and symptom type. Am J Psychiatry 157:816–818, 2000 10784477 10.1176/appi.ajp.157.5.816

[B3] Buitelaar JK, Montgomery SA, van Zwieten-Boot BJ: Attention deficit hyperactivity disorder: Guidelines for investigating efficacy of pharmacological intervention. Eur Neuropsychopharmacol 13:297–304, 2003 12888190 10.1016/s0924-977x(03)00047-6

[B4] Buitelaar JK, Wilens TE, Zhang S, Ning Y, Feldman PD: Comparison of symptomatic versus functional changes in children and adolescents with ADHD during randomized, double-blind treatment with psychostimulants, atomoxetine, or placebo. J Child Psychol Psychiatry 50:335–342, 2009 19309330 10.1111/j.1469-7610.2008.01960.x

[B5] Busner J, Targum SD: The Clinical Global Impressions scale: Applying a research tool in clinical practice. Psychiatry (Edgmont) 4:28–37, 2007 PMC2880930 20526405

[B6] Busner J, Targum SD, Miller DS: The Clinical Global Impressions scale: Errors in understanding and use. Compr Psychiatry 50:257–262, 2009 19374971 10.1016/j.comppsych.2008.08.005

[B7] Choi SW, Schalet B, Cook KF, Cella D: Establishing a common metric for depressive symptoms: Linking the BDI-II, CES-D, and PHQ-9 to PROMIS depression. Psychol Assess 26:513–527, 2014 24548149 10.1037/a0035768 PMC5515387

[B8] Cook KF, Schalet BD, Kallen MA, Rutsohn JP, Cella D: Establishing a common metric for self-reported pain: Linking BPI Pain Interference and SF-36 Bodily Pain Subscale scores to the PROMIS Pain Interference metric. Qual Life Res 24:2305–2318, 2015 25894063 10.1007/s11136-015-0987-6 PMC4567433

[B9] Cutler AJ, Brams M, Bukstein O, Mattingly G, McBurnett K, White C, Rubin J: Response/remission with guanfacine extended-release and psychostimulants in children and adolescents with attention-deficit/hyperactivity disorder. J Am Acad Child Adolesc Psychiatry 53:1092–1101, 2014 25245353 10.1016/j.jaac.2014.08.001

[B10] DuPaul GJ, Power TJ, Anastopoulos AD, Reid R: ADHD Rating Scale-5 for Children and Adolescents: Checklists, Norms, and Clinical Interpretation. New York, Guilford Publications, 2016

[B11] Faries DE, Yalcin I, Harder D, Heiligenstein JH: Validation of the ADHD Rating Scale as a clinician administered and scored instrument. J Atten Disord 5:107–115, 2001

[B12] Forkmann T, Scherer A, Boecker M, Pawelzik M, Jostes R, Gauggel S: The Clinical Global Impression scale and the influence of patient or staff perspective on outcome. BMC Psychiatry 11:83, 2011 21569566 10.1186/1471-244X-11-83 PMC3118175

[B13] Francx W, Zwiers MP, Mennes M, Oosterlaan J, Heslenfeld D, Hoekstra PJ, Hartman CA, Franke B, Faraone SV, O'Dwyer L, Buitelaar JK: White matter microstructure and developmental improvement of hyperactive/impulsive symptoms in attention-deficit/hyperactivity disorder. J Child Psychol Psychiatry 56:1289–1297, 2015 25581343 10.1111/jcpp.12379 PMC4499023

[B14] Franke B, Michelini G, Asherson P, Banaschewski T, Bilbow A, Buitelaar JK, Cormand B, Faraone SV, Ginsberg Y, Haavik J, Kuntsi J, Larsson H, Lesch KP, Ramos-Quiroga JA, Rethelyi JM, Ribases M, Reif A: Live fast, die young? A review on the developmental trajectories of ADHD across the lifespan. Eur Neuropsychopharmacol 28:1059–1088, 2018 30195575 10.1016/j.euroneuro.2018.08.001 PMC6379245

[B15] Gajria K, Kosinski M, Sikirica V, Huss M, Livote E, Reilly K, Dittmann RW, Erder MH: Psychometric validation of the Weiss Functional Impairment Rating Scale-Parent Report Form in children and adolescents with attention-deficit/hyperactivity disorder. Health Qual Life Outcomes 13:184, 2015 26577642 10.1186/s12955-015-0379-1 PMC4650258

[B16] Goodman DW, Faraone SV, Adler LA, Dirks B, Hamdani M, Weisler RH: Interpreting ADHD rating scale scores: Linking ADHD rating scale scores and CGI levels in two randomized controlled trials of lisdexamfetamine dimesylate in ADHD. Prim Psychiatry 17:44–52, 2010

[B17] Guy W: ECDEU Assessment Manual for Psychopharmacology. Rockville, MD, U.S. Department of Health, Education, and Welfare, Public Health Service, Alcohol, Drug Abuse, and Mental Health Administration, National Institute of Mental Health, Psychopharmacology Research Branch, Division of Extramural Research Programs, 1976

[B18] Haro JM, Kamath S, Ochoa S, Novick D, Rele K, Fargas A, Rodriguez M, Rele R, Orta J, Kharbeng A: The Clinical Global Impression-Schizophrenia scale: A simple instrument to measure the diversity of symptoms present in schizophrenia. Acta Psychiatr Scand 107:16–23, 2003 10.1034/j.1600-0447.107.s416.5.x 12755850

[B19] Hodgkins P, Lloyd A, Erder MH, Setyawan J, Weiss MD, Sasane R, Nafees B: Estimating minimal important differences for several scales assessing function and quality of life in patients with attention-deficit/hyperactivity disorder. CNS Spectr 22:31–40, 2017 27535815 10.1017/S1092852916000353

[B20] Jacobson NS, Truax P: Clinical significance: A statistical approach to defining meaningful change in psychotherapy research. J Consult Clin Psychol 59:12–19, 1991 2002127 10.1037//0022-006x.59.1.12

[B21] Kay SR: Positive-negative symptom assessment in schizophrenia: Psychometric issues and scale comparison. Psychiatr Q 61:163–178, 1990 2075220 10.1007/BF01064966

[B22] Kelsey D, Sumner CR, Casat CD, Coury DL, Quintana H, Saylor K, Sutton VK, Gonzalez J, Malcolm SK, Schuh KJ, Allen AJ: Once-daily atomoxetine treatment for children with attention-deficit/hyperactivity disorder, including an assessment of evening and morning behavior: A double-blind, placebo-controlled trial. Pediatrics 113:e1–e8, 2004 15231966 10.1542/peds.114.1.e1

[B23] Kemner JE, Starr HL, Ciccone PE, Hooper-Wood CG, Crockett RS: Outcomes of OROS methylphenidate compared with atomoxetine in children with ADHD: A multicenter, randomized prospective study. Adv Ther 22:498–512, 2005 16418159 10.1007/BF02849870

[B24] Kolen MJ, Brennan RL (eds): Observed score equating using the random groups design. In: Test Equating, Scaling, and Linking. New York, NY, Springer, 2014, pp. 29–63

[B25] Leon AC, Shear MK, Klerman GL, Portera L, Rosenbaum JF, Goldenberg I: A comparison of symptom determinants of patient and clinician global ratings in patients with panic disorder and depression. J Clin Psychopharmacol 13:327–331, 1993 8227491

[B26] Lepping P, Whittington R, Sambhi RS, Lane S, Poole R, Leucht S, Cuijpers P, McCabe R, Waheed W: Clinical relevance of findings in trials of CBT for depression. Eur Psychiatry 45:207–211, 2017 28957788 10.1016/j.eurpsy.2017.07.003

[B27] Leucht S, Engel RR: The relative sensitivity of the Clinical Global Impressions Scale and the Brief Psychiatric Rating Scale in antipsychotic drug trials. Neuropsychopharmacology 31:406–412, 2006 16123745 10.1038/sj.npp.1300873

[B28] Leucht S, Fennema H, Engel RR, Kaspers-Janssen M, Szegedi A: Translating the HAM-D into the MADRS and vice versa with equipercentile linking. J Affect Disord 226:326–331, 2018 29031182 10.1016/j.jad.2017.09.042

[B29] Leucht S, Kane JM, Etschel E, Kissling W, Hamann J, Engel RR: Linking the PANSS, BPRS, and CGI: Clinical implications. Neuropsychopharmacology 31:2318–2325, 2006 16823384 10.1038/sj.npp.1301147

[B30] Leucht S, Kane JM, Kissling W, Hamann J, Etschel E, Engel R: Clinical implications of brief psychiatric rating scale scores. Br J Psychiatry 187:366–371, 2005 16199797 10.1192/bjp.187.4.366

[B31] Levine SZ, Rabinowitz J, Engel R, Etschel E, Leucht S: Extrapolation between measures of symptom severity and change: An examination of the PANSS and CGI. Schizophr Res 98:318–322, 2008 17949948 10.1016/j.schres.2007.09.006

[B32] Michelson D, Allen AJ, Busner J, Casat CD, Dunn D, Kratochvil C, Newcorn JH, Sallee FR, Sangal RB, Saylor K, West S, Kelsey D, Wernicke J, Trapp NJ, Harder D: Once-daily atomoxetine treatment for children and adolescents with attention deficit hyperactivity disorder: A randomized, placebo-controlled study. Am J Psychiatry 159:1896–1901, 2002 12411225 10.1176/appi.ajp.159.11.1896

[B33] Namiki S, Takegami M, Kakehi Y, Suzukamo Y, Fukuhara S, Arai Y: Analysis linking UCLA PCI with expanded prostate cancer index composite: An evaluation of health related quality of life in Japanese men with localized prostate cancer. J Urol 178:473–477; discussion 477, 2007 17561164 10.1016/j.juro.2007.03.113

[B34] Nasser A, Hull JT, Chowdhry F, Adewole T, Liranso T, Marcus R, Schwabe S: Extended-release viloxazine (SPN-812) 200 mg or 400 mg for the treatment of ADHD in adolescents: Topline results of a phase 3, randomized, double-blind, placebo-controlled study (P302). In: 32nd Annual Psych Congress. San Diego CA, 2019a

[B35] Nasser A, Liranso T, Adewole T, Fry N, Hull JT, Chowdhry F, Busse GD, Melyan Z, Cutler AJ, Findling RL, Schwabe S. Once-daily 200-mg and 400-mg SPN-812 in the treatment of ADHD in school-age children: A phase 3 randomized controlled trial. Clin Ther. In press 10.1016/j.clinthera.2021.01.027 33750646

[B36] Nasser A, Liranso T, Adewole T, Fry N, Hull JT, Chowdhry F, Busse GD, Melyan Z, Cutler AJ, Findling RL, Schwabe S: A phase 3 placebo-controlled trial of once-daily 400-mg and 600-mg SPN-812 (viloxazine extended-release) in adolescents with ADHD. Psychopharm Bull 51:1–22, 2021 PMC8146561 34092822

[B37] Nasser A, Liranso T, Adewole T, Fry N, Hull JT, Chowdhry F, Busse GD, Cutler AJ, Jones NJ, Findling RL, Schwabe S: A phase 3, randomized, placebo-controlled trial to assess the efficacy and safety of once-daily SPN-812 (viloxazine extended release) in the treatment of ADHD in school-age children. Clin Ther 42:1452–1466, 2020 32723670 10.1016/j.clinthera.2020.05.021

[B38] Newcorn JH, Sutton VK, Zhang S, Wilens T, Kratochvil C, Emslie GJ, D'Souza D N, Schuh LM, Allen AJ: Characteristics of placebo responders in pediatric clinical trials of attention-deficit/hyperactivity disorder. J Am Acad Child Adolesc Psychiatry 48:1165–1172, 2009 19858759 10.1097/CHI.0b013e3181bc730d

[B39] Safren SA, Sprich SE, Cooper-Vince C, Knouse LE, Lerner JA: Life impairments in adults with medication-treated ADHD. J Atten Disord 13:524–531, 2010 19395647 10.1177/1087054709332460 PMC3652876

[B40] Shea JA, Norcini JJ: Equating. In: Licensure Testing: Purposes, Procedures, and Practices. Edited by Impara JC. Lincoln, NE, Buros Center for Testing, 1995, pp. 253–287

[B41] Spencer T, Biederman J, Wilens T, Doyle R, Surman C, Prince J, Mick E, Aleardi M, Herzig K, Faraone S: A large, double-blind, randomized clinical trial of methylphenidate in the treatment of adults with attention-deficit/hyperactivity disorder. Biol Psychiatry 57:456–463, 2005 15737659 10.1016/j.biopsych.2004.11.043

[B42] Spencer T, Biederman J, Wilens T, Faraone SV, Prince J, Gerard K, Doyle R, Parekh A, Kagan J, Bearman SK: Efficacy of mixed amphetamine salts compound in adults with attention-deficit/hyperactivity disorder. Arch Gen Psychiatry 58:775–782, 2001 11483144 10.1001/archpsyc.58.8.775

[B43] Sprich SE, Safren SA, Finkelstein D, Remmert JE, Hammerness P: A randomized controlled trial of cognitive behavioral therapy for ADHD in medication-treated adolescents. J Child Psychol Psychiatry 57:1218–1226, 2016 26990084 10.1111/jcpp.12549 PMC5026858

[B44] Steele M, Jensen PS, Quinn DMP: Remission versus response as the goal of therapy in ADHD: A new standard for the field? Clin Ther 28:1892–1908, 2006 17213010 10.1016/j.clinthera.2006.11.006

[B45] Thompson T, Lloyd A, Joseph A, Weiss M: The Weiss Functional Impairment Rating Scale-Parent Form for assessing ADHD: Evaluating diagnostic accuracy and determining optimal thresholds using ROC analysis. Qual Life Res 26:1879–1885, 2017 28220338 10.1007/s11136-017-1514-8 PMC5486894

[B46] Weiss M, Childress A, Mattingly G, Nordbrock E, Kupper RJ, Adjei AL: Relationship between symptomatic and functional improvement and remission in a treatment response to stimulant trial. J Child Adolesc Psychopharmacol 28:521–529, 2018 30036076 10.1089/cap.2017.0166 PMC6201781

[B47] Weiss M, Childress A, Nordbrock E, Adjei AL, Kupper RJ, Mattingly G: Characteristics of ADHD symptom response/remission in a clinical trial of methylphenidate extended release. J Clin Med 8:461, 2019 10.3390/jcm8040461 PMC6517933 30959790

[B48] Wilens T, Biederman J, Spencer T: Attention deficit/hyperactivity disorder across the lifespan. Annu Rev Med 53:113–131, 2002 11818466 10.1146/annurev.med.53.082901.103945

[B49] Wilens TE, Biederman J, Spencer TJ, Bostic J, Prince J, Monuteaux MC, Soriano J, Fine C, Abrams A, Rater M: A pilot controlled clinical trial of ABT-418, a cholinergic agonist, in the treatment of adults with attention deficit hyperactivity disorder. Am J Psychiatry 156:1931–1937, 1999 10588407 10.1176/ajp.156.12.1931

[B50] Wilens TE, Spencer TJ, Biederman J, Girard K, Doyle R, Prince J, Polisner D, Solhkhah R, Comeau S, Monuteaux MC: A controlled clinical trial of bupropion for attention deficit hyperactivity disorder in adults. Am J Psychiatry 158:282–288, 2001 11156812 10.1176/appi.ajp.158.2.282

[B51] Yu C, Garcia-Olivares J, Candler S, Schwabe S, Maletic V: New insights into the mechanism of action of viloxazine: Serotonin and norepinephrine modulating properties. J Exp Pharmacol 12:285–300, 2020 32943948 10.2147/JEP.S256586 PMC7473988

[B52] Zhang S, Faries DE, Vowles M, Michelson D: ADHD Rating Scale IV: Psychometric properties from a multinational study as a clinician-administered instrument. Int J Methods Psychiatr Res 14:186–201, 2005 16395872 10.1002/mpr.7 PMC6878282

