# Supplementary material for: Translating Attention-Deficit/Hyperactivity Disorder Rating Scale-5 and Weiss Functional Impairment Rating Scale-Parent Effectiveness Scores into Clinical Global Impressions Clinical Significance Levels in Four Randomized Clinical Trials of SPN-812 (Viloxazine Extended-Release) in Children and Adolescents with Attention-Deficit/Hyperactivity Disorder
Source: J Child Adolesc Psychopharmacol. 2021 Apr 16;31(3):214–26. doi: 10.1089/cap.2020.0148 (PMC8066343; doi:10.1089/cap.2020.0148)
Supplement: Supplemental data [file Supp_TableS1.docx]

Table S1: Distribution of baseline ADHD-RS-5 Total scores and CGI-S levels used to generate the link function.

| Patient Population | CGI-S | N | Mean (SD) | Quartiles | Range |
| --- | --- | --- | --- | --- | --- |
| **Overall** | 4 - Moderately ill | 556 | 38.4 (7.00) | (33, 38, 44) | 18 to 54 |
|  | 5 - Markedly ill | 626 | 44.1 (6.39) | (40, 45, 49) | 11 to 54 |
|  | 6 - Severely ill | 162 | 48.2 (5.54) | (45, 50, 52) | 22 to 54 |
|  | 7 - Extremely ill | 10 | 52.9 (1.10) | (52, 53, 54) | 51 to 54 |
| **Children** | 4 - Moderately ill | 266 | 40.0 (6.58) | (35, 40, 45) | 23 to 54 |
|  | 5 - Markedly ill | 384 | 45.4 (5.72) | (42, 46, 50) | 28 to 54 |
|  | 6 - Severely ill | 103 | 49.6 (4.11) | (48, 51, 52) | 36 to 54 |
|  | 7 - Extremely ill | 8 | 53.1 (0.99) | (52, 54, 54) | 52 to 54 |
| **Adolescents** | 4 - Moderately ill | 290 | 36.8 (7.03) | (31, 36, 41) | 18 to 54 |
|  | 5 - Markedly ill | 242 | 42.1 (6.89) | (38, 43, 47) | 11 to 54 |
|  | 6 - Severely ill | 59 | 45.7 (6.77) | (42, 47, 51) | 22 to 54 |
|  | 7 - Extremely ill | 2 | 52.0 (1.41) | (51, 52, 53) | 51 to 53 |
