# Supplementary material for: Translating Attention-Deficit/Hyperactivity Disorder Rating Scale-5 and Weiss Functional Impairment Rating Scale-Parent Effectiveness Scores into Clinical Global Impressions Clinical Significance Levels in Four Randomized Clinical Trials of SPN-812 (Viloxazine Extended-Release) in Children and Adolescents with Attention-Deficit/Hyperactivity Disorder
Source: J Child Adolesc Psychopharmacol. 2021 Apr 16;31(3):214–26. doi: 10.1089/cap.2020.0148 (PMC8066343; doi:10.1089/cap.2020.0148)
Supplement: Supplemental data [file Supp_TableS2.docx]

Table S2: Distribution of End-of-Study Absolute Change from Baseline ADHD-RS-5 Total scores and CGI-I levels used to generate the link function.

| Patient Population | CGI-I | N | Mean (SD) | Quartiles | Range |
| --- | --- | --- | --- | --- | --- |
| **Overall** | 1 - Very much improved | 260 | -33.2 (8.88) | (-41, -34, -27) | -54 to -7 |
|  | 2 - Much improved | 329 | -21.3 (8.44) | (-27, -21, -15) | -48 to 10 |
|  | 3 - Minimally improved | 301 | -11.8 (8.18) | (-15, -11, -7) | -48 to 16 |
|  | 4 - No change | 431 | -2.7 (7.27) | (-6, -1, 1) | -38 to 22 |
|  | 5 - Minimally worse | 26 | 2.5 (7.02) | (2, 4, 7) | -23 to 11 |
|  | 6 - Much worse | 6 | 4.7 (7.26) | (0, 2, 8) | -1 to 18 |
|  | 7 - Very much worse | 1 | 15.0 | 15 | 15 |
| **Children** | 1 - Very much improved | 134 | -34.6 (8.49) | (-41, -35, -28) | -54 to -10 |
|  | 2 - Much improved | 188 | -21.3 (8.62) | (-27, -21, -15) | -48 to 7 |
|  | 3 - Minimally improved | 164 | -12.7 (8.08) | (-16, -11, -7) | -48 to 5 |
|  | 4 - No change | 255 | -2.5 (6.82) | (-5, -1, 1) | -35 to 18 |
|  | 5 - Minimally worse | 14 | 2.2 (8.07) | (2, 4, 7) | -23 to 8 |
|  | 6 - Much worse | 5 | 2.0 (3.54) | (0, 1, 2) | -1 to 8 |
|  | 7 - Very much worse | 1 | 15.0 | 15 | 15 |
| **Adolescents** | 1 - Very much improved | 126 | -31.7 (9.06) | (-39, -32, -25) | -52 to -7 |
|  | 2 - Much improved | 141 | -21.4 (8.22) | (-26, -21, -16) | -45 to 10 |
|  | 3 - Minimally improved | 137 | -10.8 (8.20) | (-15, -10, -6) | -38 to 16 |
|  | 4 - No change | 176 | -2.9 (7.90) | (-6, -2, 1) | -38 to 22 |
|  | 5 - Minimally worse | 12 | 2.8 (5.89) | (-2, 4, 7) | -7 to 11 |
|  | 6 - Much worse | 1 | 18.0 | 18 | 18 |
