# Supplementary material for: Translating Attention-Deficit/Hyperactivity Disorder Rating Scale-5 and Weiss Functional Impairment Rating Scale-Parent Effectiveness Scores into Clinical Global Impressions Clinical Significance Levels in Four Randomized Clinical Trials of SPN-812 (Viloxazine Extended-Release) in Children and Adolescents with Attention-Deficit/Hyperactivity Disorder
Source: J Child Adolesc Psychopharmacol. 2021 Apr 16;31(3):214–26. doi: 10.1089/cap.2020.0148 (PMC8066343; doi:10.1089/cap.2020.0148)
Supplement: Supplemental data [file Supp_FigS2.docx]

Figure S2. Link Functions for WFIRS-P scores and CGI/CGI Levels by Treatment.

Link functions for WFIRS-P and CGI scores did not differ between pediatric patients receiving SPN-812 (red triangles) or placebo (blue lines). Shaded bands represent 95% confidence intervals. For clarity, the upper range on panel C was cut off at 100% (see Figure 4C in the main text for the full range), and some points were omitted without changing the shape of the line: included on panel A is every 10^th^ point, panel B is every 15^th^ point, panel C is every 6^th^ point. **(A)** Baseline scores, **(B)** absolute change from baseline at end of study, **(C)** percent change from baseline at end of study.
