# Supplementary material for: Translating Attention-Deficit/Hyperactivity Disorder Rating Scale-5 and Weiss Functional Impairment Rating Scale-Parent Effectiveness Scores into Clinical Global Impressions Clinical Significance Levels in Four Randomized Clinical Trials of SPN-812 (Viloxazine Extended-Release) in Children and Adolescents with Attention-Deficit/Hyperactivity Disorder
Source: J Child Adolesc Psychopharmacol. 2021 Apr 16;31(3):214–26. doi: 10.1089/cap.2020.0148 (PMC8066343; doi:10.1089/cap.2020.0148)
Supplement: Supplemental data [file Supp_TableS3.docx]

Table S3: Distribution of End-of-Study Percent Change from Baseline ADHD-RS-5 Total scores and CGI-I levels used to generate the link function.

| Patient Population | CGI-I | N | Mean (SD) | Quartiles | Range |
| --- | --- | --- | --- | --- | --- |
| **Overall** | 1 - Very much improved | 260 | -77.7 (13.98) | (-87, -80, -71) | -100 to -17 |
|  | 2 - Much improved | 329 | -51.4 (17.85) | (-64, -52, -41) | -100 to 23 |
|  | 3 - Minimally improved | 301 | -28.1 (18.47) | (-38, -26, -17) | -89 to 55 |
|  | 4 - No change | 431 | -5.8 (18.68) | (-13, -3, 2) | -81 to 110 |
|  | 5 - Minimally worse | 26 | 8.2 (20.30) | (4, 9, 20) | -49 to 61 |
|  | 6 - Much worse | 6 | 14.0 (24.56) | (0, 3, 18) | -2 to 62 |
|  | 7 - Very much worse | 1 | 38.0 | 38 | 38 |
| **Children** | 1 - Very much improved | 134 | -78.2 (12.76) | (-86, -81, -71) | -100 to -29 |
|  | 2 - Much improved | 188 | -49.0 (17.76) | (-62, -49, -37) | -100 to 15 |
|  | 3 - Minimally improved | 164 | -28.9 (17.44) | (-37, -25, -18) | -89 to 16 |
|  | 4 - No change | 255 | -5.3 (15.84) | (-11, -3, 2) | -80 to 55 |
|  | 5 - Minimally worse | 14 | 6.4 (18.60) | (4, 9, 20) | -49 to 24 |
|  | 6 - Much worse | 5 | 4.4 (7.92) | (0, 2, 4) | -2 to 18 |
|  | 7 - Very much worse | 1 | 38.0 | 38 | 38 |
| **Adolescents** | 1 - Very much improved | 126 | -77.2 (15.20) | (-88, -80, -70) | -100 to -17 |
|  | 2 - Much improved | 141 | -54.6 (17.52) | (-67, -54, -45) | -94 to 23 |
|  | 3 - Minimally improved | 137 | -27.2 (19.66) | (-40, -27, -14) | -78 to 55 |
|  | 4 - No change | 176 | -6.4 (22.20) | (-17, -5, 2) | -81 to 110 |
|  | 5 - Minimally worse | 12 | 10.3 (22.78) | (-5, 10, 18) | -25 to 61 |
|  | 6 - Much worse | 1 | 62.0 | 62 | 62 |
