# Supplementary material for: Translating Attention-Deficit/Hyperactivity Disorder Rating Scale-5 and Weiss Functional Impairment Rating Scale-Parent Effectiveness Scores into Clinical Global Impressions Clinical Significance Levels in Four Randomized Clinical Trials of SPN-812 (Viloxazine Extended-Release) in Children and Adolescents with Attention-Deficit/Hyperactivity Disorder
Source: J Child Adolesc Psychopharmacol. 2021 Apr 16;31(3):214–26. doi: 10.1089/cap.2020.0148 (PMC8066343; doi:10.1089/cap.2020.0148)
Supplement: Supplemental data [file Supp_TableS4.docx]

Table S4: Distribution of baseline WFIRS-P Total Average scores and CGI-S levels used to generate the link function.

| Patient Population | CGI-S / CGI-I | N | Mean (SD) | Quartiles | Range |
| --- | --- | --- | --- | --- | --- |
| **Overall** | 4 - Moderately ill | 522 | 0.93 (0.47) | (0.6, 0.9, 1.2) | 0.1 to 2.9 |
|  | 5 - Markedly ill | 571 | 1.10 (0.44) | (0.8, 1.1, 1.4) | 0.1 to 2.8 |
|  | 6 - Severely ill | 149 | 1.26 (0.47) | (0.9, 1.2, 1.6) | 0.2 to 2.6 |
|  | 7 - Extremely ill | 10 | 1.54 (0.42) | (1.3, 1.5, 1.8) | 1.0 to 2.5 |
| **Children** | 4 - Moderately ill | 247 | 0.96 (0.47) | (0.6, 0.9, 1.3) | 0.1 to 2.9 |
|  | 5 - Markedly ill | 351 | 1.15 (0.44) | (0.8, 1.1, 1.5) | 0.1 to 2.8 |
|  | 6 - Severely ill | 94 | 1.30 (0.42) | (0.9, 1.3, 1.6) | 0.6 to 2.5 |
|  | 7 - Extremely ill | 8 | 1.56 (0.47) | (1.3, 1.4, 1.8) | 1.0 to 2.5 |
| **Adolescents** | 4 - Moderately ill | 275 | 0.90 (0.47) | (0.5, 0.8, 1.2) | 0.1 to 2.6 |
|  | 5 - Markedly ill | 220 | 1.03 (0.43) | (0.7, 1.0, 1.3) | 0.1 to 2.2 |
|  | 6 - Severely ill | 55 | 1.20 (0.54) | (0.8, 1.2, 1.5) | 0.2 to 2.6 |
|  | 7 - Extremely ill | 2 | 1.48 (0.06) | (1.4, 1.5, 1.5) | 1.4 to 1.5 |
