# Supplementary material for: Translating Attention-Deficit/Hyperactivity Disorder Rating Scale-5 and Weiss Functional Impairment Rating Scale-Parent Effectiveness Scores into Clinical Global Impressions Clinical Significance Levels in Four Randomized Clinical Trials of SPN-812 (Viloxazine Extended-Release) in Children and Adolescents with Attention-Deficit/Hyperactivity Disorder
Source: J Child Adolesc Psychopharmacol. 2021 Apr 16;31(3):214–26. doi: 10.1089/cap.2020.0148 (PMC8066343; doi:10.1089/cap.2020.0148)
Supplement: Supplemental data [file Supp_TableS5.docx]

Table S5: Distribution of End-of-Study Absolute Change from Baseline WFIRS-P Total Average scores and CGI-I levels used to generate the link function.

| Patient Population | CGI-S / CGI-I | N | Mean (SD) | Quartiles | Range |
| --- | --- | --- | --- | --- | --- |
| **Overall** | 1 - Very much improved | 255 | -0.57 (0.49) | (-0.9, -0.5, -0.2) | -2.0 to 1.2 |
|  | 2 - Much improved | 303 | -0.34 (0.40) | (-0.6, -0.3, -0.1) | -2.2 to 1.4 |
|  | 3 - Minimally improved | 276 | -0.27 (0.43) | (-0.5, -0.2, -0.0) | -2.5 to 1.0 |
|  | 4 - No change | 391 | -0.09 (0.34) | (-0.3, -0.1, 0.1) | -1.6 to 1.1 |
|  | 5 - Minimally worse | 22 | -0.01 (0.26) | (-0.2, -0.1, 0.2) | -0.5 to 0.7 |
|  | 6 - Much worse | 5 | 0.17 (0.18) | (0.1, 0.1, 0.2) | -0.0 to 0.4 |
| **Children** | 1 - Very much improved | 130 | -0.60 (0.48) | (-0.9, -0.5, -0.3) | -1.9 to 1.0 |
|  | 2 - Much improved | 169 | -0.41 (0.44) | (-0.6, -0.4, -0.1) | -2.2 to 1.4 |
|  | 3 - Minimally improved | 152 | -0.31 (0.45) | (-0.5, -0.3, -0.1) | -2.5 to 1.0 |
|  | 4 - No change | 232 | -0.11 (0.33) | (-0.3, -0.1, 0.1) | -1.2 to 0.9 |
|  | 5 - Minimally worse | 13 | 0.04 (0.26) | (-0.1, -0.0, 0.0) | -0.2 to 0.7 |
|  | 6 - Much worse | 4 | 0.18 (0.21) | (0.0, 0.2, 0.3) | -0.0 to 0.4 |
| **Adolescents** | 1 - Very much improved | 125 | -0.53 (0.51) | (-0.9, -0.5, -0.2) | -2.0 to 1.2 |
|  | 2 - Much improved | 134 | -0.25 (0.33) | (-0.4, -0.2, -0.1) | -1.2 to 0.8 |
|  | 3 - Minimally improved | 124 | -0.23 (0.39) | (-0.5, -0.2, 0.0) | -1.2 to 0.8 |
|  | 4 - No change | 159 | -0.05 (0.36) | (-0.2, -0.0, 0.1) | -1.6 to 1.1 |
|  | 5 - Minimally worse | 9 | -0.07 (0.26) | (-0.2, -0.2, 0.2) | -0.5 to 0.2 |
|  | 6 - Much worse | 1 | 0.13 | 0.1 | 0.1 |
