# Supplementary material for: Translating Attention-Deficit/Hyperactivity Disorder Rating Scale-5 and Weiss Functional Impairment Rating Scale-Parent Effectiveness Scores into Clinical Global Impressions Clinical Significance Levels in Four Randomized Clinical Trials of SPN-812 (Viloxazine Extended-Release) in Children and Adolescents with Attention-Deficit/Hyperactivity Disorder
Source: J Child Adolesc Psychopharmacol. 2021 Apr 16;31(3):214–26. doi: 10.1089/cap.2020.0148 (PMC8066343; doi:10.1089/cap.2020.0148)
Supplement: Supplemental data [file Supp_TableS6.docx]

Table S6: Distribution of End-of-Study Percent Change from Baseline WFIRS-P Total Average scores and CGI-I levels used to generate the link function.

| Patient Population | CGI-S / CGI-I | N | Mean (SD) | Quartiles | Range |
| --- | --- | --- | --- | --- | --- |
| **Overall** | 1 - Very much improved | 255 | -49.11 (40.53) | (-76.0, -59.0, -33.0) | -100 to 250 |
|  | 2 - Much improved | 303 | -28.14 (43.28) | (-55.0, -33.0, -13.0) | -100 to 410 |
|  | 3 - Minimally improved | 276 | -20.46 (36.12) | (-44.5, -24.0, -6.0) | -97 to 163 |
|  | 4 - No change | 391 | -4.04 (35.19) | (-26.0, -7.0, 14.0) | -97 to 168 |
|  | 5 - Minimally worse | 22 | -1.18 (28.79) | (-19.0, -3.5, 19.0) | -52 to 52 |
|  | 6 - Much worse | 5 | 12.80 (11.48) | (11.0, 13.0, 16.0) | -4 to 28 |
| **Children** | 1 - Very much improved | 130 | -51.99 (34.44) | (-77.0, -59.0, -37.0) | -100 to 86 |
|  | 2 - Much improved | 169 | -33.20 (38.39) | (-58.0, -38.0, -17.0) | -100 to 200 |
|  | 3 - Minimally improved | 152 | -23.74 (33.97) | (-45.0, -25.5, -10.5) | -97 to 163 |
|  | 4 - No change | 232 | -6.16 (35.44) | (-27.5, -9.0, 9.5) | -97 to 168 |
|  | 5 - Minimally worse | 13 | 5.38 (24.24) | (-8.0, -3.0, 1.0) | -19 to 52 |
|  | 6 - Much worse | 4 | 12.00 (13.09) | (3.5, 12.0, 20.5) | -4 to 28 |
| **Adolescents** | 1 - Very much improved | 125 | -46.11 (45.97) | (-75.0, -59.0, -27.0) | -100 to 250 |
|  | 2 - Much improved | 134 | -21.76 (48.16) | (-44.0, -27.0, -9.0) | -86 to 410 |
|  | 3 - Minimally improved | 124 | -16.43 (38.35) | (-43.0, -23.0, 2.0) | -76 to 139 |
|  | 4 - No change | 159 | -0.95 (34.69) | (-25.0, -3.0, 17.0) | -85 to 129 |
|  | 5 - Minimally worse | 9 | -10.67 (33.51) | (-38.0, -26.0, 19.0) | -52 to 39 |
|  | 6 - Much worse | 1 | 16.00 | 16.0 | 16 |
